# Mosquito identification and haemosporidian parasites detection in the enclosure of the African penguins (*Spheniscus demersus*) at the SANBI zoological garden

**DOI:** 10.1016/j.ijppaw.2020.08.004

**Published:** 2020-09-03

**Authors:** Moeti O. Taioe, Mafanela C. Mnisi, Mamohale Chaisi, Monica Mwale, Ndzalama Mabunda, Veronica Phetla, Oriel M.M. Thekisoe

**Affiliations:** a Foundational Research and Services, South African National Biodiversity Institute, National Zoological Gardens, PO Box 754, Pretoria, 0001, South Africa; b Unit for Environmental Sciences and Management, North West University, Private Bag X6001, Potchefstroom, 2520, South Africa

**Keywords:** African penguin enclosure, haemosporidian parasites, mosquitoes, National Zoological Gardens

## Abstract

The National Zoological Gardens (NZG) is a facility of the South African National Biodiversity Institute (SANBI) and the largest zoo in southern Africa. Among the 9000 captive animals kept by the NZG, is the endangered African penguin (*Spheniscus demersus*). There have been several post-mortem reports on deaths of penguins in the NZG due to haemosporidian infections, however, the haemosporidian lineages involved and possible insect vector are unknown. Haemosporidians are apicomplexan parasites that infect vertebrates through blood-sucking dipteran insects. Therefore, the current study aimed to identify mosquitoes that are potential vectors found within the African penguin enclosure as well as to detect the haemosporidian parasites from these insects using nested-PCR and real-time PCR (qPCR) analyses. Mosquito samples were collected using an overnight UV-light trap setup for 3 months. From the 65 pooled samples representing 325 mosquitoes, morphological and molecular analysis showed that *Culex pipiens* (52.31%) was the dominant species followed by *Cx.theileri* (30.77%) and *Cx. quinquefasciatus* (16.92%). Nested-PCR detected parasite DNA of *Leucocytozoon* sp. and *Plasmodium* sp. The *Cx. pipiens* had the highest minimum infection rate (MIR) of 5.88% by nested-PCR and 9.41% by qPCR whilst *Cx. quinquefasciatus* had MIR of 3.64% in both assays and no haemosporidian parasites were detected from *Cx.theileri.* One *Cx. pipiens* sample had a co-infection of both *Plasmodium* sp. and *Leucocytozoon* sp. detected by nested-PCR. These findings suggest that effective control measures for blood-sucking dipteran insects is required at the NZG and more studies should be conducted to determine the actual prevalence of these haemosporidian parasites among other bird species within NZG.

## Highlights

• Three mosquito species, namely, *Culex pipiens*, *Cx. quinquefasciatus* and *Cx.* *theileri* occur in the African penguin enclosure at the SANBI zoological garden.
• DNA of *Leucocytozoon* sp. and *Plasmodium* sp. was detected from the *Culex pipiens* and *Cx. quinquefasciatus* mosquitoes.
• None of the haemosporidian parasite DNA was detected from the *Cx.* *theileri.*
• The detection of *Leucocytozoon* sp. DNA from *Culex* mosquitoes is uncommon as they are not documented vectors of these parasites.
• Data of this study raises need to assess the vectorial capacity for *Leucocytozoon* sp. and *Culex* mosquitoes.

## Graphical abstract

**Figure:**
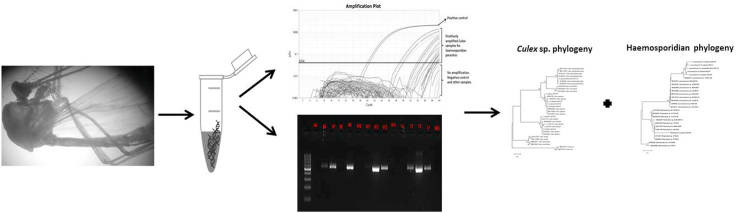

## 1 Introduction

The genus *Culex* is widely reported as an excellent vector for *Plasmodium* parasites (Njabo et al., 2009; Santiago-Alarcon et al., 2012; Valkiūnas and Iezhova, 2018). There are 763 species in this genus occurring in all zoogeographical regions except Antarctica, with 136 species from 8 subgenera recorded from the Afrotropics (Coetzee, 2017). Despite several reports of the transmission of *Plasmodium* parasites by *Culex* mosquitoes, their diversity, abundance and their possible role in the transmission of haemosporidian parasites in the National Zoological Garden remains unknown.

Haemosporidians (Sporozoa: Haemosporida) are obligatory parasites belonging to the phylum Apicomplexa that infect birds, amphibians, reptiles as well as mammals and are transmitted by blood-sucking dipteran insects (Valkiūnas, 2005). The avian haemosporidian parasites are divided into four main genera: *Leucocytozoon*, *Haemoproteus*, *Plasmodium* with a cosmopolitan distribution, whilst the genus *Fallisia* is confined to the Neotropical region (Hellgren et al., 2004). They are characterized by heteroxenous life cycles, with the dipteran insect vector as the definitive host (sexual stages and sporogony) and the vertebrate animal as the intermediate host (asexual stages and development of gametocytes) (Hellgren et al., 2004; Mantilla et al., 2016). These parasites infect both domestic and wild avian populations with clinical symptoms varying from a pale mucous membrane, dyspnea, lethargy and preacute death (Robinson, 2009). Symptoms in penguins vary depending on the haemosporidian parasite involved, age (chick, juvenile or adult) as well as their locality (captive vs. wild). It has been reported that captive penguins infected with avian malaria (*Plasmodium* sp.) may not display any clinical symptoms, however, typical signs can include loss of appetite, weight loss, respiratory distress, lethargy, weakness, pale mucous membranes, isolation from the group, vomiting, regurgitation following force-feeding and greenish stool (Grilo et al., 2016). Lesions associated with severe acute infections include hepatomegaly, splenomegaly, lung congestion and hydropericardium due to the presence of tissue meronts in major organs [spleen, lungs, liver and heart] (Grilo et al., 2016; Vanstreels et al., 2015, 2016, 2017). Infections from *Leucocytozoon* sp. have also shown to be fatal among chick and juvenile penguins and less severe in adults (Vanstreels et al., 2016). Studies on infections from *Haemoproteus* sp. in penguins have reported that all samples that tested positive for the parasite had no external clinical signs of illness suggesting that the infection might be abortive (Vanstreels et al., 2016). However, a study by Cannell et al. (2013) on wild juvenile penguins (*Eudyptula minor*) has demonstrated that infections from *Haemoproteus* sp. can be fatal.

The traditional technique used to identify these parasites has always been microscopic examination of blood slides, but, it is less sensitive during low parasitemia, requires skilled personnel and can be laborious (Hellgren et al., 2004; Braga et al., 2011; Bell et al., 2015). Recently, molecular techniques such as the nested-PCR and real-time PCR (qPCR) targeting the haemosporidian *cytochrome b* gene have made it feasible for the accurate and rapid detection of avian haemosporidian parasites (Braga et al., 2011; Clark et al., 2014). However, molecular techniques are prone to false positives and multiple PCRs must be performed to detect closely related haemosporidian parasites (Valkiūnas et al., 2008). Furthermore, Bernotienė et al. (2016) reported that various PCR assays underestimate the actual diversity of haemosporidian parasites as they may fail to detect mixed infections. But, studies by Valkiūnas et al. (2006, 2008; 2016) have demonstrated that the sensitivity of both microscopic and molecular techniques can yield similar results when detecting haemosporidian parasites from blood samples, provided that the blood slides and DNA used are of good quality. Furthermore, the development of the MalAvi database of avian haemosporidian lineages (http://130.235.244.92/Malavi/), has enabled scientists to record global distribution patterns of the haemosporidian parasite lineages, the avian-vertebrate host as well as the genetic information of the dipteran vector from various geographical locations and it is freely available (Bensch et al., 2009; Clark et al., 2014).

The National Zoological Gardens (NZG) is a facility of the South African National Biodiversity Institute (SANBI) and the largest zoo in southern Africa. Between November 2018 and January 2020, seven adult female penguins have died as a result of avian malaria and its associated illness. The unpublished veterinary report from the zoo hospital did indicate that the responsible haemosporidian parasites were transmitted by mosquito vectors but could not establish which mosquito species was responsible. As a result, the current study aimed to identify mosquito vectors found within the African penguin enclosure using both morphological and molecular techniques as well as to determine the occurrence of the haemosporidian parasites harbored by these insects using nested-PCR complemented by the qPCR assay. Furthermore, this study sought to determine the phylogenetic relationships of the haemosporidian lineages with published lineages from GenBank and the MalAvi databases.

## 2 Materials and methods

### 2.1 Study site

**Fig. 1:**
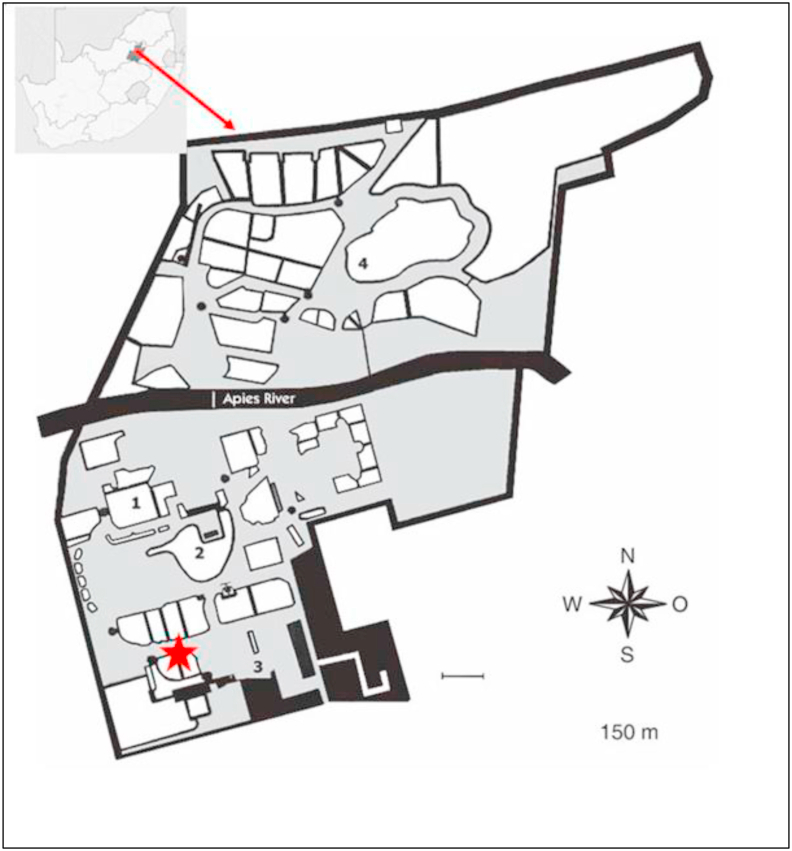
Map of South Africa showing the National Zoological Gardens (NZG). The red star indicates where the African penguin enclosure is located and where mosquito samples were collected (Labuschagne et al., 2008). (For interpretation of the references to color in this figure legend, the reader is referred to the Web version of this article.)

The National Zoological Gardens (NZG) is located in central Pretoria (25°44′18″ S; 28°11′21″ E) in the Gauteng Province, South Africa (Fig. 1). It is approximately 80 ha and forms part of the Gauteng Shale Mountain Bushveld biome with a landscape that is relatively flat and the second half located on slopes of the Magaliesberg Mountain range with the Apies River separating the two halves. Among the 9000 captive animals kept by the NZG, is the endangered African penguin (*Spheniscus demersus*). Within the African penguin enclosure, there are currently 28 adult penguins with 11 breeding pairs (13 male and 15 female) and no juveniles. Inside the enclosure, there is a pool of still water as shown in supplementary file (Fig. S1. A) which is an ideal breeding site for mosquitoes and the vegetation is composed of small shrubs and small trees to make the enclosure as natural as possible. There are small populations of wild birds ranging from the Egyptian goose (*Alopochen aegyptiaca*), spur-winged goose (*Plectropterus gambensis*), African sacred ibis (*Threskiornis aethiopicus*), hadada ibis (*Bostrychia hagedash*) and large populations of wild feral (*Columba guinea*) and speckled (*C. livia*) pigeons that frequent the zoo. There are three types of insect repellents within the penguin enclosure, a liquid repellent that uses electricity to release the liquid in the air, single-bulb and double-bulb UV-light killer zappers placed in the middle of the feeding site and 3 outdoor mosquito deterrent fans (Fig. S1. B - D). Data downloaded from the national weather services (https://www.weathersa.co.za), showed that the average rainfall during the sampling period (November 2019–January 2020) was ≥110 mm with December having the highest average precipitation at 150 mm whilst, the average temperature was 16 °C minimum and 28 °C maximum with January being the warmest month with an average maximum temperature of 31 °C.

### 2.2 Sample collection

**Fig. 2:**
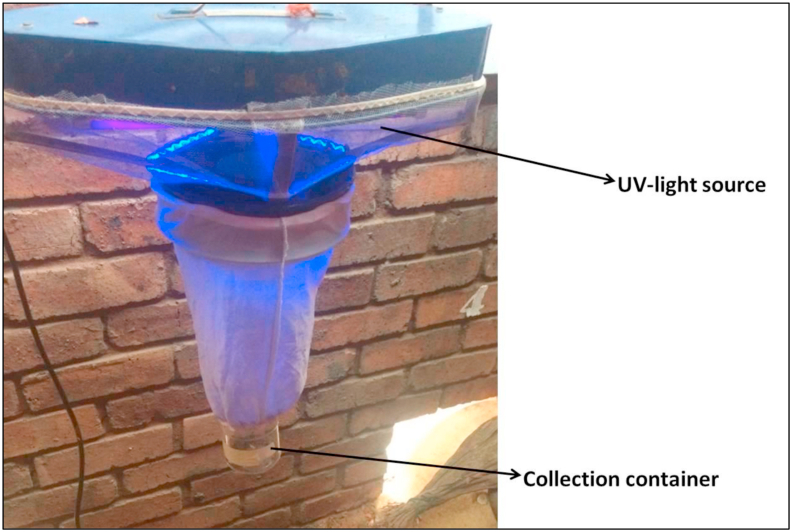
The single UV-light trap used to collect mosquito samples.

Ethical approval was granted by the SANBI-NZG Research Ethics and Scientific Committee (Project number: SANBI/NZG/RES/P19-06). Mosquito samples were collected using a single UV-light trap (Fig. 2) located behind the roosting nests of the breeding pairs. The UV-light trap was setup for 3 months (switched on at dusk and off in the morning) (between November 2019 and January 2020) and monitored every 48 h to collect the captured specimens. Captured specimens that continued to actively fly in the collection containers were exposed to a temperature of −4 °C in the freezer for euthanization and then stored in 70% ethanol.

### 2.3 Mosquito identification

Captured mosquito samples were initially sorted into morphospecies. They were then identified to species level, using a dissecting microscope based on unique morphological features from the head, thorax as well as the wing venation and color patterns on the abdomen and legs as described by Jupp (1996).

### 2.4 DNA extraction

Prior to DNA extraction, none of the mosquito samples were dissected nor checked for engorged females. The ZymoBIOMICS™ Miniprep (Zymo Research Corporation, USA) was used to extract DNA from mosquitoes. Initially, a whole mosquito from each species was homogenized in the ZR BashingBead™ lysis tube containing 750 μl ZymoBIOMICS™ Lysis Solution and 20 μl Proteinase K (20 μg/μl) and incubated for 1 h at 56 °C. Thereafter, the ZR BashingBead™ lysis tubes were horizontally secured on a beat beater at 1600 rpm for 1 h at 56 °C. Subsequently, the manufacturer's protocol was followed for complete DNA extraction. The DNA concentrations from the extracted samples were then quantified using a Nanodrop™ ND-1000 UV–Visible (Life Technologies Inc. USA) and stored at −20 °C. Samples used for the detection of haemosporidian parasites were pooled into groups of 5 specimens of each species per sample and DNA was extracted in the same manner described above.

### 2.5 PCR amplification and sequencing

#### 2.5.1 Identification of mosquito by PCR

The PCR amplification targeting the mosquito's *cytochrome oxidase I* (*COI*) gene region was conducted using primers that were described by Folmer et al. (1994). The PCR reaction mixture had a final volume of 25 μl containing 12.5 μl of 2× *Taq* DNA Polymerase Master Mix RED™ [Tris-HCl pH 8·05, (NH_4_)_2_SO_4,_ 3 mM MgCl_2,_ 0.2% Tween®20, 0.4 mM of each dNTP, Ampliqon *Taq* DNA Polymerase and Inert red dye with stabilizer] (Ampliqon A/S, DK), 0.4 μM of each primer, 2 μl (~40 ng) of template DNA and double-distilled water (ddH_2_O) was added to final volume. Genomic DNA of *Tabanus par* (TP14-SA) obtained from the study conducted by Taioe et al. (2017) was used as a positive control and ddH_2_O as a negative control. The PCR conditions were set as follows: 1 step of 95 °C for 3 min, 95 °C for 15 s, 55 °C for 30 s and 72 °C for 30 s at 35 cycles and the final extension at 72 °C for 5 min. Amplicons were visualized using 1.5% agarose gel stained with SYBR Safe DNA Gel Stain (Applied Biosystems, USA) under UV light.

#### 2.5.2 Detection of haemosporidian parasites by nested PCR

Amplification of the 617 bp fragment of the *cytochrome-b* gene (*cyt-b*) region for the detection of avian haemosporidian parasites (*Leucocytozoon* sp. *Haemoproteus* sp. and *Plasmodium* sp.) was conducted by nested-PCR using primers published by Hellgren et al. (2004). The primary PCR reaction had a final volume of 25 μl containing 12.5 μl of 2× *Taq* DNA Polymerase Master Mix RED™ with similar volumes as described above. Synthetic DNA (gBlocks®) [Integrated DNA Technologies (IDT), USA] of *Plasmodium relictum* (GenBank accession no: NC012426) and *Leucocytozoon fringillinarun* (GenBank accession no: FJ168564) were used as positive controls and ddH_2_O as a negative control. The PCR conditions were set as follows: 1 cycle of 95 °C for 5 min, 95 °C for 30 s, 50 °C for 30 s and 72 °C for 45 s at 20 cycles for the primary PCR and 35 cycles for the nested-PCR with the last extension at 72 °C for 10 min. The nested-PCR was carried out with 1 μl of the primary PCR product. All PCR products were visualized using 1.5% agarose gel stained with SYBR Safe DNA Gel Stain (Applied Biosystems, USA) and visualized under UV light.

### 2.6 Detection of haemosporidian by quantitative PCR (qPCR)

Quantitative PCR (qPCR) analysis was conducted to confirm observations made from nested-PCR analysis. We used primers published by Bell et al. (2015). Amplification of the 182 bp of the conserved *ribosomal DNA* (*rDNA*) fragment of the haemosporidian parasites was performed with a QuantStudio™12 K Flex System (Applied Biosystems, USA) platform. The reaction mixture contained 3 μl (~60 ng) of template DNA, 0.4 μM of each primer. A total of 7.5 μl of PowerUp™ SYBR® Green Master Mix [SYBR® Green Dye, Dual-Lock™ Taq DNA polymerase enzyme, dNTPs with dUTP/dTTP blend, heat-labile UDG, ROX passive reference dye, and optimized buffer components] (Applied Biosystems, USA) and ddH_2_O to make a final reaction volume of 15 μl. The amplification conditions were set as follows: 50 °C for 2 min, 95 °C for 2 min, then 40 cycles at 95 °C for 15 s, and 53 °C for 15 s and 72 °C for 1 min. The melting curve settings were at 95 °C for 15 s, 60 °C for 1 min and 95 °C for 15 s. The threshold of the amplification target was set at 0.04. Synthetic DNA fragment (gBlocks®) of *P. relictum* (as indicated above) was used as a positive control, and ddH_2_O as a negative control.

### 2.7 Sequencing and nucleotide basic local alignment search tool (BLASTn)

All positively amplified products from both *COI-*PCR and *cyt-b* nested PCR were cleaned-up and purified prior to cycle sequencing using a combination of alkaline phosphatase FastAP™ and exonuclease ExoSap™ (Applied Biosystems, USA) enzymes, following the manufacturer's protocol. Sequencing was conducted using BigDye Terminator™ v3.1 cycle sequencing kit according to the manufacture's protocol (Applied Biosystems, USA). Subsequently, the product was loaded into a 96 well plate and ran on the ABI PRISM 3500™ automated DNA sequencer (Applied Biosystems, USA) for electrophoresis, SeqScape™ and Genescan software™ (Applied Biosystems, USA) were used for base-calling and scoring. Analysis of the data was done using the Sequence Analyzer software version 1.7·1 (Developed by Will Gilbert, http://informagen.com/SA/). Retrieved gene sequences were edited using MEGAX (Kumar et al., 2018). All edited *COI* and *cyt-b* sequences were subsequently subjected to BLASTn (www.ncbi.nlm.nih.gov/blast/) whereby the gene sequences with 90–100% similarity match score were considered as significant. Furthermore, *cyt-b* sequences were subjected to a BLAST analysis in the MalAvi database (http://130.235.244.92/Malavi/) to determine the haemosporidian lineages, sequences differing by one or more bases from the known parasite lineages were described as unique/new lineages (Bensch et al., 2009).

### 2.8 Phylogenetic analyses

To determine the phylogenetic relationship of the mosquitoes collected from the NZG obtained *cytochrome oxidase I* (*COI*) gene sequences along with those downloaded sequences from the NCBI database, were aligned by ClustalW with default parameters on MEGAX for multiple and pairwise sequence alignments (Kumar et al., 2018). MEGAX was used to edit and trim as well as to determine the appropriate evolutionary model to construct the maximum likelihood (ML) tree. The best model selected was the Tamura 3-parameter with Gamma Distributed (G) rates among sites set at 5.00 (+*G*, parameter = 0.0500) and 1000 bootstrap replicates to evaluate the tree topology (Kumar et al., 2018; Nei and Kumar, 2000). There was a total of 31 *COI* gene sequences with a total length of 633 bp which were used to construct the tree whereby *Lutzia* sp. (GenBank accession no: MK271011 and MK271012) were used as outgroups.

The *cyt-b* gene sequences were aligned, edited and trimmed as mentioned above. The sequence alignment was conducted with *cyt-b* gene sequences from this study as well as those downloaded from the MalAvi and NCBI databases. Maximum likelihood tree topology with 1000 cycles of bootstrap support was constructed on MEGAX (Kumar et al., 2018). Tamura 3-parameter with Gamma Distributed (G) rates among sites set at 5.00 (+*G*, parameter = 0.4125) was used to construct the ML tree. There was a total of 35 *cyt-b* gene sequences with a total length of 438 bp which were used to construct the tree and *Haemoproteus* sp. (GenBank accession no: KU562203 and MG018628) were used as outgroups.

### 2.9 Statistical analysis

Relative abundance of mosquitoes was calculated as the number of a species divided by the total number of sampled mosquitoes, multiplied by 100. Pearson's Chi square test (χ^2^) at 95% confidence level was used to determine the significance in the overall abundance of the sampled mosquitoes over the sampling period. However, samples that had no prominent morphological identification features (missing head, legs or wings) and by-catch insects were excluded from the analysis. The prevalence of haemosporidian parasites detected by both assays was calculated considering a fixed pool size and 100% test for specificity and sensitivity (Veiga et al., 2018). Thereafter, Epitools bySergeant, 2018 was used to determine the pooled prevalence estimates with the exact confidence limits at 95% (*p* < 0.05) from the sampled mosquitoes. The minimum infection rate (MIR), representing the number of positive pools relative to the mosquitoes tested was subsequently calculated as MIR (%) = n_(PCR positive pools)_/n_(total analyzed mosquitoes)_ × 100 (Ejiri et al., 2008; Schoener et al., 2017; Ventim et al., 2012). Confidence of the mean at 95% intervals was used to determine the prevalence of the detected haemosporidian parasites. Thereafter, student *t-*test assuming equal variance at 95% was used to determine the significant difference between the MIRs of both assays.

## 3 Results

### 3.1 Identification and relative abundance of mosquitoes

A total of 325 mosquitoes were collected from the African penguin enclosure at the NZG over a period of three months and grouped into 65 pools. Morphological analysis revealed that *Culex pipiens* was the most abundant species throughout the sampling period with the relative abundance of 52.31%, followed by *Cx.* *theileri* with 30.77% and *Cx. quinquefasciatus* with 16.92% (Table 1). There was no significant difference in the overall abundance of the sampled mosquitoes throughout the sampling period (χ^2^ = 5.365, df = 4, *p* = 0.99). The unique descriptive features used to identify these mosquitoes are provided in Supplementary file (Figs. S2–S4).

**Table 1:** Abundance of mosquito species within the National Zoological Gardens (NZG) penguin enclosure.

Mosquito Species	Nov-19 (n)	Dec-19 (n)	Jan-20 (n)	Number of samples	Relative abundance (%)
*Cx.theileri*	6.15 (20)	18.46 (60)	6.15 (20)	100	30.77
*Cx. pipiens*	10.77 (35)	27.69 (90)	13.58 (45)	170	52.31
*Cx. quinquefasciatus*	1.54 (5)	10.77 (35)	4.62 (15)	55	16.92
**Total**	**18.46 (60)**	**56.92 (185)**	**24.62 (80)**	**325**	

### 3.2 Phylogeny of mosquitoes

**Fig. 3:**
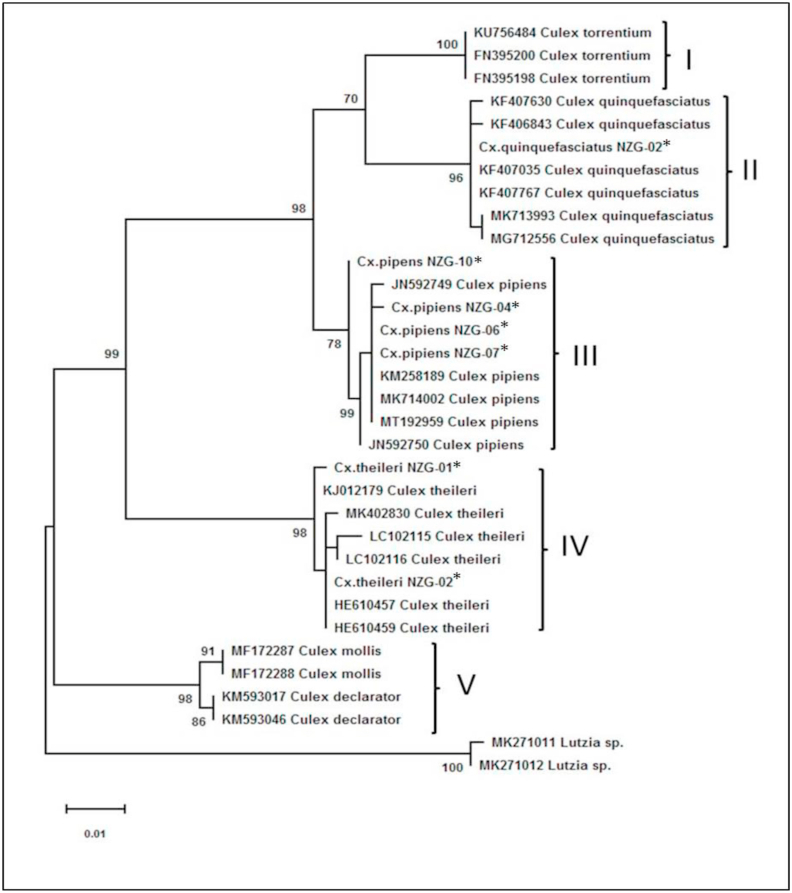
Maximum likelihood tree for *Culex* species showing the 5 clades representing 5 subgroups. Clade I is the Trifilatus Subgroup (Mattingly and Rageau, 1958) for *Cx.torrentium*; Clade II and III are the Pipiens Complex; Clade IV the Theileri Subgroup (Sirivanakarn, 1976) for *Cx.theileri*; and Clade V is the Tarsalis (Edwards, 1932) for *Cx. declaratory* and Apicinus Subgroups (Edwards, 1932) for *Cx. mollis*. *Lutzia* sp. used as outgroups. Sequences from this study are indicated by asterisks (*).

The *COI* gene sequences obtained from this study were deposited into NCBI GenBank under the accession numbers MT506033 to MT506038, MT512680 and MT512681. The maximum likelihood tree topology (Fig. 3) had high nodal bootstrap (>70%) support values and confirmed the phylogenetic placement of the *Culex* species collected from the NZG African penguin enclosure when aligned with publicly available sequences of *Culex* sp. from various countries. A total of 5 monophyletic clades, representing 5 previously published subgroups (Apicinus Subgroup, Tarsalis Subgroup, Trifilatus Subgroup, Theileri Subgroup and the Pipiens Complex) were observed from the tree topology with the log likelihood of −1355.42.

### 3.3 Detection of haemosporidian parasites from mosquito DNA by nested and qPCR

The pooled mosquito samples tested positive for *Leucocytozoon* sp. and *Plasmodium* sp. by nested-PCR (Fig. S5), whilst qPCR amplification peaks do not differentiate between infections from the different genera (Fig. S6). *Culex pipiens and Cx. quinquefasciatus,* both from the Pipiens Complex, were the only mosquito species that tested positive for the presence of haemosporidian parasites. The overall prevalence of the detected haemosporidian parasites by nested-PCR was 4.0% (95% CI: 2.07–6.89%, n = 12 positive pool samples), whilst the overall prevalence detected by qPCR was 6.3% (95% CI: 3.73–9.77%, n = 18 positive pool samples). The prevalence of *Leucocytozoon* sp. by nested PCR was 3.6% (95% CI: 1.82–6.43%, n = 11 positive pool samples), whereas the prevalence of *Plasmodium* sp. by nested PCR was 0.31% (95% CI: −0.30–0.92%, n = 1 positive pool sample). The minimum infection rate (MIR) for the respective mosquitoes by nested-PCR was as follows: *Cx. pipiens* at 5.88% and *Cx. quinquefasciatus* was 3.64%, whilst, the MIR by qPCR was 9.41% for *Cx. pipiens* and 3.64% for *Cx. quinquefasciatus* (Table 2). There was no significant difference in the MIRs between nested-PCR and qPCR (t = −0.89; df = 2, *p* = 0.23). Lastly, there was a co-infection of *Leucocytozoon* sp. and *Plasmodium* sp. in one pooled sample of *Cx. pipiens* sample [*Cx. pipiens_*NZG-06 (M6)], detected by nested-PCR and this is indicated as sample M6 on the agarose gel (Fig. S5).

**Table 2:** Minimum infection rate (MIR) for the prevalence of haemosporidian parasites in sampled mosquitoes.

Mosquito species	No of collected mosquitoes	No of pooled samples	No of +ve by nested PCR	No of +ve by qPCR	MIR for nested PCR (%)	MIR for qPCR (%)
*Cx.theileri*	100	20	0	0	0	0
*Cx. pipiens*	170	34	10	16	5.88	9.41
*Cx. quinquefasciatus*	55	11	2	2	3.64	3.64
**Total**	**325**	**65**	**12**	**18**	**9.52**	**13.05**

(+ve): is an abbreviation for number of samples that tested positive in either nested PCR or qPCR.

### 3.4 Phylogeny of haemosporidian parasites

**Fig. 4:**
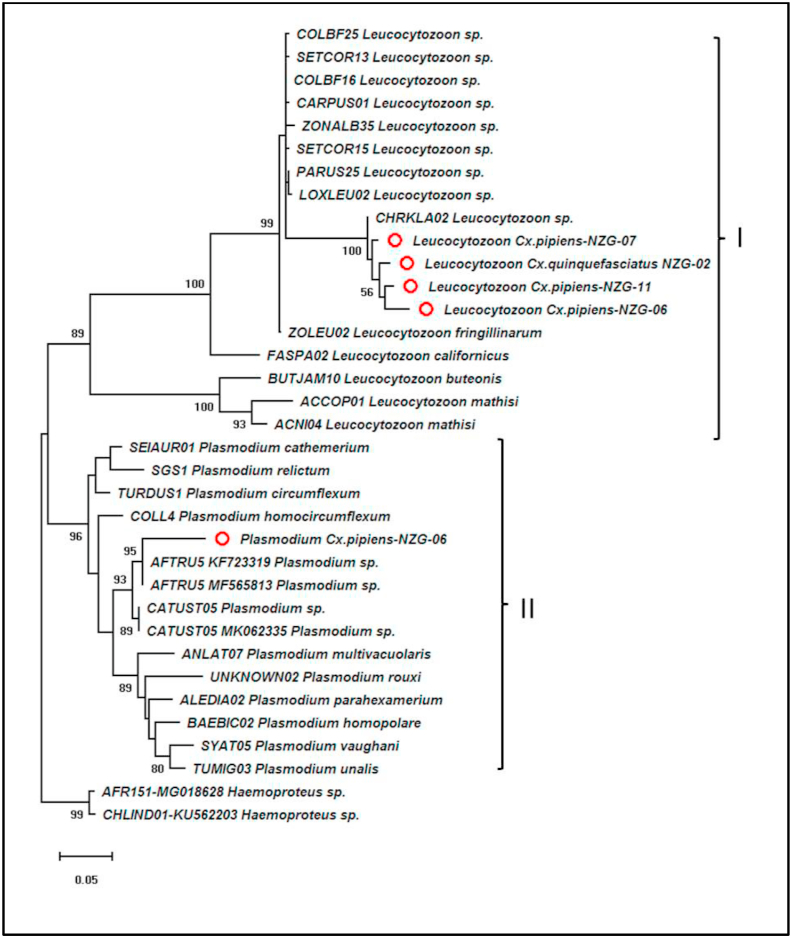
Maximum likelihood tree showing the clustering of *Leucocytozoon* sp. (Clade I) and *Plasmodium* sp. (Clade II) with *Haemoproteus* sp. as outgroup. Sequences from this study are highlighted with red circles. (For interpretation of the references to color in this figure legend, the reader is referred to the Web version of this article.)

The BLASTn analyses of the obtained *Leucocytozoon* sp. and *Plasmodium* sp. sequences together with published sequences from GenBank and MalAvi databases did not reveal any identical sequences (100%), indicating that the *Leucocytozoon* sp. and *Plasmodium* sp. obtained from this study are novel lineages. The maximum likelihood tree topology used to infer the phylogeny of the detected haemosporidian parasites showed two major monophyletic clades (*Plasmodium* sp. and *Leucocytozoon* sp.) that had high nodal support (Fig. 4). In clade I, the *Leucocytozoon* sp. sequences generated from this study clustered together and formed a paraphyletic clade with *Leucocytozoon* lineage CHRKLA02 (accession no: MH492307) with 100% bootstrap support. All other *Leucocytozoon* lineages from the MalAvi and NCBI databases clustered together with the exception of *L. fringillinarum* (lineage ZOLEU02). Similar observations were made on Clade II where the *Plasmodium* sp. sequence obtained from this study clustered with two *Plasmodium* AFTRU5 lineage sequences (accession no: KF723319 and MF565813) and formed a paraphyletic clade with other *Plasmodium* lineages from the databases. These observations do support the suggestion that generated sequences from this study are indeed novel lineages. This was however, expected in the case of *Leucocytozoon* sp. sequences as this haemosporidian parasite is not known to infect mosquito vectors.

## 4 Discussion

The identification of insect vectors and the evaluation of their infection rates to predict the disease prevalence are crucial in any study of vector-borne diseases (Inci et al., 2012; Ferraguti et al., 2013). The current study identified potential avian malaria vectors and the prevalence of their haemosporidian parasites within the African penguin enclosure using morphological and molecular techniques. To our knowledge, this is the first baseline report on the occurrence of haemosporidian parasites from mosquitoes within the NZG. A similar study was conducted by Labuschagne et al. (2008) that focused on the abundance and diversity of *Culicoides* biting midges using only morphological features and did not detect possible pathogens that might be harbored by these flies.

Morphological and molecular analysis indicated that *Culex pipiens*, *Cx.* *theileri* and *Cx. quinquefasciatus* were the abundant mosquito species within the African penguin enclosure during the sampling period. These findings are comparable with those reported by Okanga et al. (2013) in the Western Cape, South Africa as well as other parts of the world, where members of the *Pipiens* Complex were reported as the most abundant mosquito species during their sampling periods (Ejiri et al., 2008, 2011; Ferraguti et al., 2013; Morchón et al., 2013; Schoener et al., 2017; Lam-Phua et al., 2019). There was no significant difference in the relative abundance of the sampled mosquitoes with December month having the highest number of catches. This was consistent with observations made by Santa-Ana et al. (2006) in the Madeira Island of Portugal, where they recorded a significant increase in the number of *Cx. pipiens* and *Cx.* *theileri* caught in the same month due to the an increase in rainfall.

The resolved *COI* phylogeny was able to separate *Cx. pipiens* and *Cx. quinquefasciatus* clades with strong nodal support. This is in contrast to observations made by Tahir et al. (2016), where they could not separate *Cx. quinquefasciatus* and *Cx. pipiens* based on their *COI* barcode sequence. However, it has been reported that the genus *Culex* is highly diverse and most species are difficult to identify on morphological basis (Tahir et al., 2016). For species morphological identification, genitalia are used to identify *Culex* males however for females, larvae are used for accurate identification and this is also dependent upon the availability of a family of siblings which have been reared from one female individual (Jupp, 1996). As such, complementing morphological analysis with *COI-*gene based barcoding is reliable in species identification. It is also essential to consider that, in most studies conducted on the abundance of mosquitoes (Ejiri et al., 2008, 2011; Inci et al., 2012; Ferraguti et al., 2013; Okanga et al., 2013; Schoener et al., 2017; Lam-Phua et al., 2019), sampling was conducted annually or seasonally which was not the situation in the current study.

The detection of *Leucocytozoon* DNA from the tested *Culex* samples is uncommon and to our knowledge this is the first time such a case is reported in southern Africa. The known biological vectors of *Leucocytozoon* sp. include black flies (Diptera: Simuliidae) and biting midges *Culicoides* (Diptera: Ceratopogonidae) (Yu and Wang, 2001; Adler and McCreadie, 2002; Valkiūnas, 2005; Jumpato et al., 2019). In addition, the sporogonic development of *Leucocytozoon* sp. lineages in mosquitoes has by no means been documented and in the current study, the *Culex* samples were not dissected and examined under a microscope to determine which developmental stages (sporozoite, ookinete or oocyst) were detected by nested-PCR. Experimental studies on the vectorial capacity of *Cx. pipiens* to transmit *Plasmodium* sp. and *Haemoproteus* sp. parasites showed that *Cx. pipiens* are competent vectors of *Plasmodium* sp. and not *Haemoproteus* sp. due to the lack of infective sporozoites in the salivary glands of the *Ochlerotatus cantans* mosquitoes tested (Gutiérrez-López et al., 2016). The authors concluded that this might be a result of an abortive sporogonic development at the oocyst stage and the same can be said about the presence of *Leucocytozoon* DNA detected from *Culex* samples. According to the MaAlvi database, the haemosporidian parasites from this study are similar to lineage CHRKLA02 for *Leucocytozoon* sp. and lineage AFTRU5 for *Plasmodium* sp. The CHRKLA02 lineage has been reported from wild avian populations of Diederik cuckoo (*Chrysococcyx caprius*) from South Africa by Chaisi et al. (2019) whilst, the AFTRU5 lineage has been reported from thrushes (Turdidae) in Western Africa by (Beadell et al., 2009) and in carrion crows (*Corvus corone*) from Germany (Schmid et al., 2017). Pooled whole mosquitoes (mixture of engorged and non-engorged female mosquitoes) were used in the current study, there is a possibility that the detection of the *Leucocytozoon* DNA might be from an infected blood meal source and not from the mosquito itself. Although uncommon the presence of *Leucocytozoon* DNA from the *Culex* mosquitoes in this study, means that the possibility of mechanical transmission cannot be ruled out.

The minimum infection rate (MIR) assumes that, when a pool tests positive, only one mosquito sample in that pool is actually infected (Inci et al., 2012; Schoener et al., 2017). Subsequently, the number of positive pools in relation to the mosquitoes tested is examined, and this relationship is generalized onto the overall mosquito population in the environment (Inci et al., 2012). The overall MIR in the current study was 9.52% by nested-PCR and 13.05% by qPCR respectively, with *Cx. pipiens* (9.41%) having the highest MIR followed by *Cx. quinquefasciatus* (3.64%). Previous research has shown that there is variation in the MIRs of different mosquito species (Ventim et al., 2012; Schoener et al., 2017). For example, *Cx. pipiens* has been shown to have MIRs ranging from 0.52%, 3.08%–43.1% in three different studies conducted in Japan (Ejiri et al., 2008, 2009; Kim et al., 2009), 1.91%–5.36% in Austria from different *Cx. pipiens* subspecies (Schoener et al., 2017), 0.04% in Portugal (Ventim et al., 2012) and 16.22% in Turkey (Inci et al., 2012), respectively. *Culex quinquefasciatus* had MIR of 1.1% in a study by Ejiri et al. (2008) which was quite less to what was observed in the current study. On the contrary, no haemosporidian parasite DNA was detected in *Cx.* *theileri* samples whilst, in previous studies the MIR varied between 0.03% (Ventim et al., 2012), 5.18% (Inci et al., 2012) and 18.8% in blood-fed *Cx.* *theileri* mosquitoes from Spain (Ferraguti et al., 2013). The variation observed in the MIRs of *Cx. pipiens* and *Cx. quinquefasciatus* may be due to difference in the preferred hosts, as it has been observed in different studies that *Cx. pipiens* is more attracted to avian hosts whereas *Cx. quinquefasciatus* are more attracted to mammalian hosts (Fryxell et al., 2014). As such, our findings are consistent with previous studies and we therefore, suggest that all three *Culex* species are potential vectors of avian malaria (*Plasmodium* sp.) parasites at the NZG despite the low prevalence reported and the absence of nested-PCR positives from the sampled *Cx.* *theileri.* Furthermore, since whole mosquito samples were used in the pools, it was not possible to assert vector competence for *Leucocytozoon* in the current study.

The overall prevalence of the detected haemosporidian parasites by nested-PCR was 4.0% and 6.3% by real-time PCR (qPCR). *Leucocytozoon* sp. had the highest prevalence at 3.60% and 0.31% for *Plasmodium* sp. In the current study, qPCR was more sensitive than nested-PCR which suggests that it has a higher detection potential for host samples with low parasitemia (Bell et al., 2015). This is in contrast to observations made by Chaisi et al. (2019), where they detected more haemosporidian parasites with nested PCR (n = 77) than qPCR (n = 64) however, the variation was not statistically significant as in the current study. The authors attributed this difference to possible sequence variations in the target region of the qPCR assay which possibly compromises the sensitivity of the assay in amplifying some parasite lineages, resulting in false negatives. Our findings are consistent with those made by Heym et al. (2019), where they detected all three parasites with *Leucocytozoon* sp. (n = 10) being more prevalent than *Plasmodium* sp. (n = 1) from mosquitoes collected from two zoological gardens in Germany. On the contrary, reports from studies where parasites were detected from mosquito vectors have mostly detected *Plasmodium* sp. with various lineages and *Haemoproteus* sp. and no *Leucocytozoon* sp. have been documented. Only studies where parasites have been detected from wild and captive avian hosts have reported on the prevalence of *Leucocytozoon* sp. with various lineages in some cases (Imura et al., 2012; Schmid et al., 2017; Jia et al., 2018; Chaisi et al., 2019; Van Hemert et al., 2019; Villar Couto et al., 2019). The detection of both *Leucocytozoon* sp. and *Plasmodium* sp. in *Cx. pipiens* were expected as they are also common in avian hosts, however, this observation may be a by-product of examining pools of mosquitoes instead of looking at individuals (Schoener et al., 2017). The lineages of the detected haemosporidian parasites were said to be novel with closest matches to CHRKLA02 lineage for *Leucocytozoon* sp. and AFTRU5 lineage for *Plasmodium* sp. from the MalAvi database. These lineages were reported from free ranging avian hosts (Beadell et al., 2009; Schmid et al., 2017; Chaisi et al., 2019) and we therefore suspect that *Cx. pipiens* from this study got infected with *Leucocytozoon* lineages by chance when feeding from an infected avian host and the infection is abortive as this parasite is biologically transmitted by black flies (Simuliidae) and biting midges (Ceratopogonidae) (Adler and McCreadie, 2019).

Studies detecting haemosporidian parasites in mosquito vectors have been conducted in other zoological gardens including Japan (Ejiri et al., 2009, 2011) and Germany (Heym et al., 2019) and they have reported that members of the *Culex pipiens* group had most infections and *Plasmodium* spp. were the most prevalent parasites harbored by these vectors. The pathogenicity of the detected haemosporidian parasites in wild and captive penguins is well documented (Grilo et al., 2016; Vanstreels et al., 2015, 2016, 2017). However, the pathogenicity of these newly derived lineages is unknown and requires urgent attention in the efforts of the NZG to conserve and protect the endangered African penguins (*Spheniscus demersus*). Currently the African penguin enclosure at the NZG has few insect repellents (Fig. S1. A - D) and requires major upgrades to prevent mosquitoes and other blood feeding insects from feeding on the captive penguin population as well as to prevent wild avian populations from coming in contact with the captive penguins as the origin of these detected *Leucocytozoon* lineages is unknown and no blood-meal analysis was conducted in the current study.

## 5 Conclusion

Factors such as rainfall, season, temperature, water quality and habitat have been shown to have an effect on the abundance and diversity of mosquitoes (Okanga et al., 2013). In efforts of preserving endangered avian species in a protected environment it is crucial to evaluate the threat posed by blood-sucking dipteran vectors, the protozoan parasites (and other pathogens) they may possibly harbor, their dispersal and distribution range as well as their preferred avian or mammalian host. In this study mosquitoes were conveniently sampled in a hot and rainy season in South Africa, we can therefore conclude that findings from this study demonstrated that (i) there are diverse mosquito species found within the African penguin enclosure at the NZG in relatively high abundance, (ii) the *Culex* species were found to harbor distinct lineages of haemosporidian parasites and (iii) *Leucocytozoon* sp. DNA detected from the tested *Culex* samples needs to be further evaluated and the vectorial capacity of these insects against various *Leucocytozoon* lineages needs to be determined because insufficient vector knowledge is an obstacle in understanding the epidemiology of haemosporidian infections in avian populations. These findings are of significance as they have pointed out potential vectors of avian malaria and related haemosporidian parasites within the NZG. This data highlights the need for improved control measures of blood feeding dipteran vectors at NZG enclosures.

## Funding

This study was made possible by multiple funds awarded to MC (SANBI Operational Core grant APCO); MM (SANBI Operational core grant APPC) and to OMMT (10.13039/501100001321National Research Foundation [NRF] Incentive grant for rated researchers [GUN94187]). The Grant holder acknowledges that opinions, findings and conclusions or recommendations expressed in any publication generated by the 10.13039/501100001321NRF supported research is that of the author(s), and that the NRF accepts no liability whatsoever in this regard.

## Declaration of competing interest

All authors contributed in the draft of this manuscript and declare no conflict of interest.
