# Supplementary material for: Mosquito identification and haemosporidian parasites detection in the enclosure of the African penguins (Spheniscus demersus) at the SANBI zoological garden
Source: Int J Parasitol Parasites Wildl. 2020 Sep 3;13:98–105. doi: 10.1016/j.ijppaw.2020.08.004 (PMC7493043; doi:10.1016/j.ijppaw.2020.08.004)
Supplement: Multimedia component 1 [file mmc1.docx]

**Supplementary files**

**
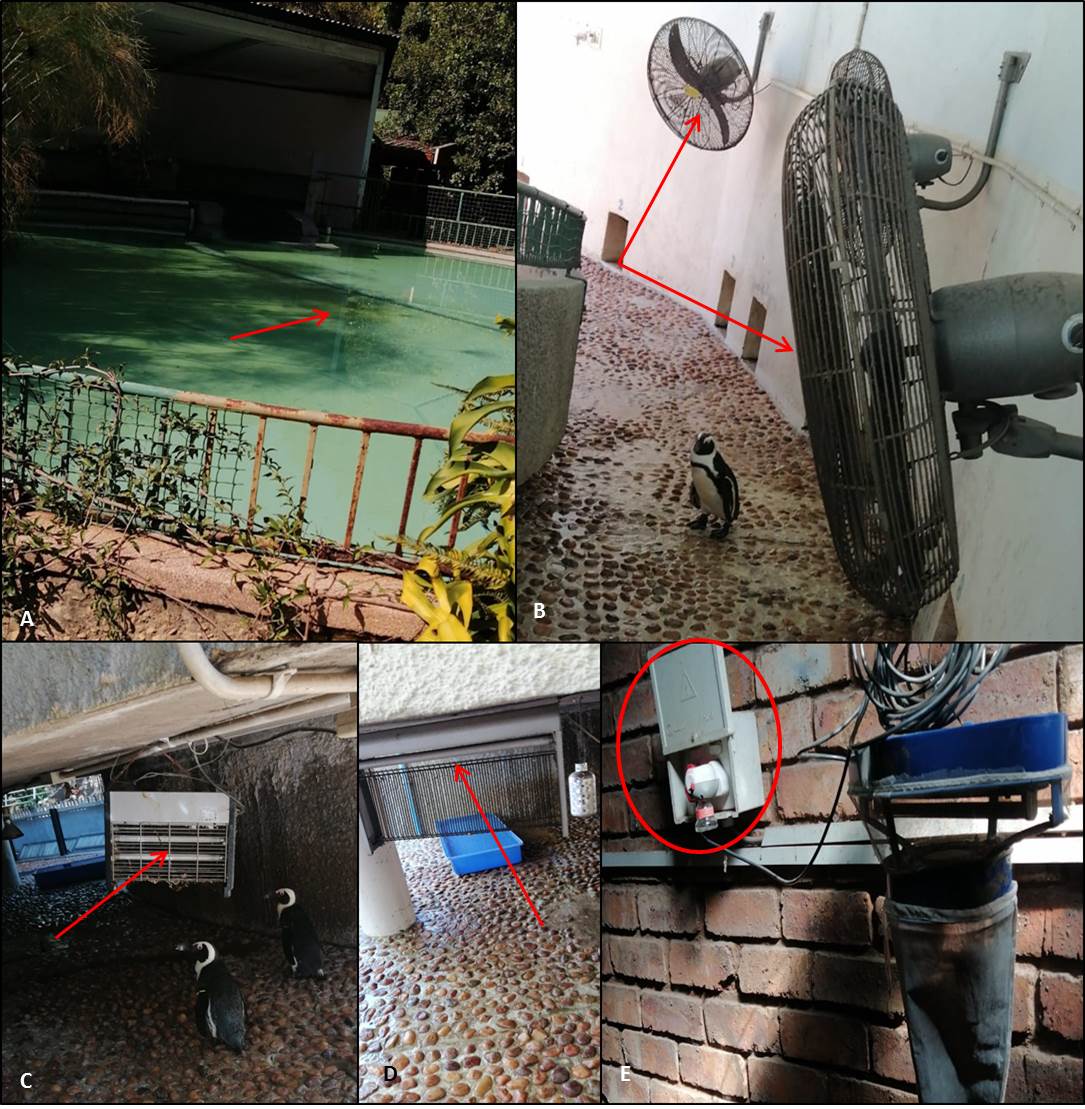
**

**Fig. S1. Images of the penguin enclosure at NZG. A -** Pool of still water within the African penguin enclosure; **B -** Three outdoor mosquito deterrent fans within the penguin enclosure; **C -** Double-bulb UV-light killer zappers placed in the middle of the feeding site; **D -** Single-bulb UV-light killer zappers; **E -** An electric liquid mosquito repellent located behind the roosting sites circled in a red circle.

**Morphological features used for mosquito identification**

The *Culex pipiens* Linnaeus, 1758 was identified with a poorly developed basal tergal bands, dark brown scutal scales and a cross vein index in wing (Jupp, 1996). The specimen had a truncated apex of the dorsal arm. The Femora and tibiae had no continuous white lines. The sterna had a pronounced dark apical band extending towards base medially; proboscis entirely dark (Fig. S2. A). The wings have short pale stripe of inner margin of costa near base (Fig. S2. B) and scutum was poorly ornate with absent prelar scales (Jupp, 1996).


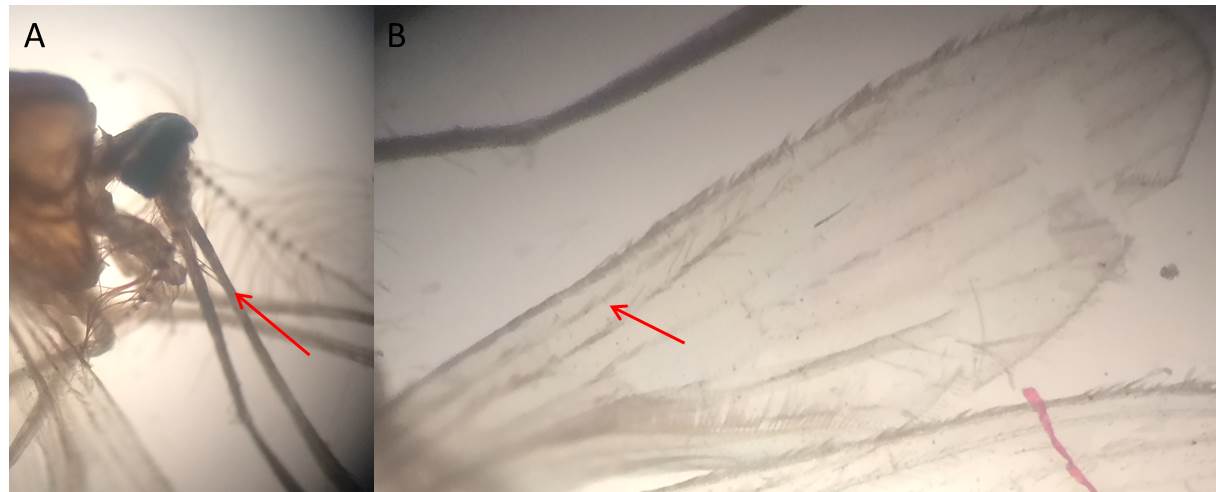


Fig. S2. Photos of the *Culex pipiens* Linnaeus, 1758. A Head and thorax of *Cx. pipiens* highlighting the dark proboscis; B: Wing venation of *Cx. pipiens* showing the pale short margin near the costa.

Features identified for *Cx. theileri* Theobald, 1903 as described by Jupp (1996) included presence of postspiracular scales. The femora and tibiae (except hidfemur) with white longitudinal line extending over their entire length and a similar line present on all first tarsomeres. The hindfemur had a dark dorsal line extending almost to bas and the anteroventral pale area continuing on distal 0.5 to reach the apex as median white line. Mesokatepisternum had a continuous row of scales on the posterior margin which is joined to prealar scales. There are 2 white stripes present ventrally on palpomeres 4-5 subapical lobe of gonocoxite with setae *d-e* consisting of a single hair-like seta with an apically flattened *f* seta (Jupp, 1996).


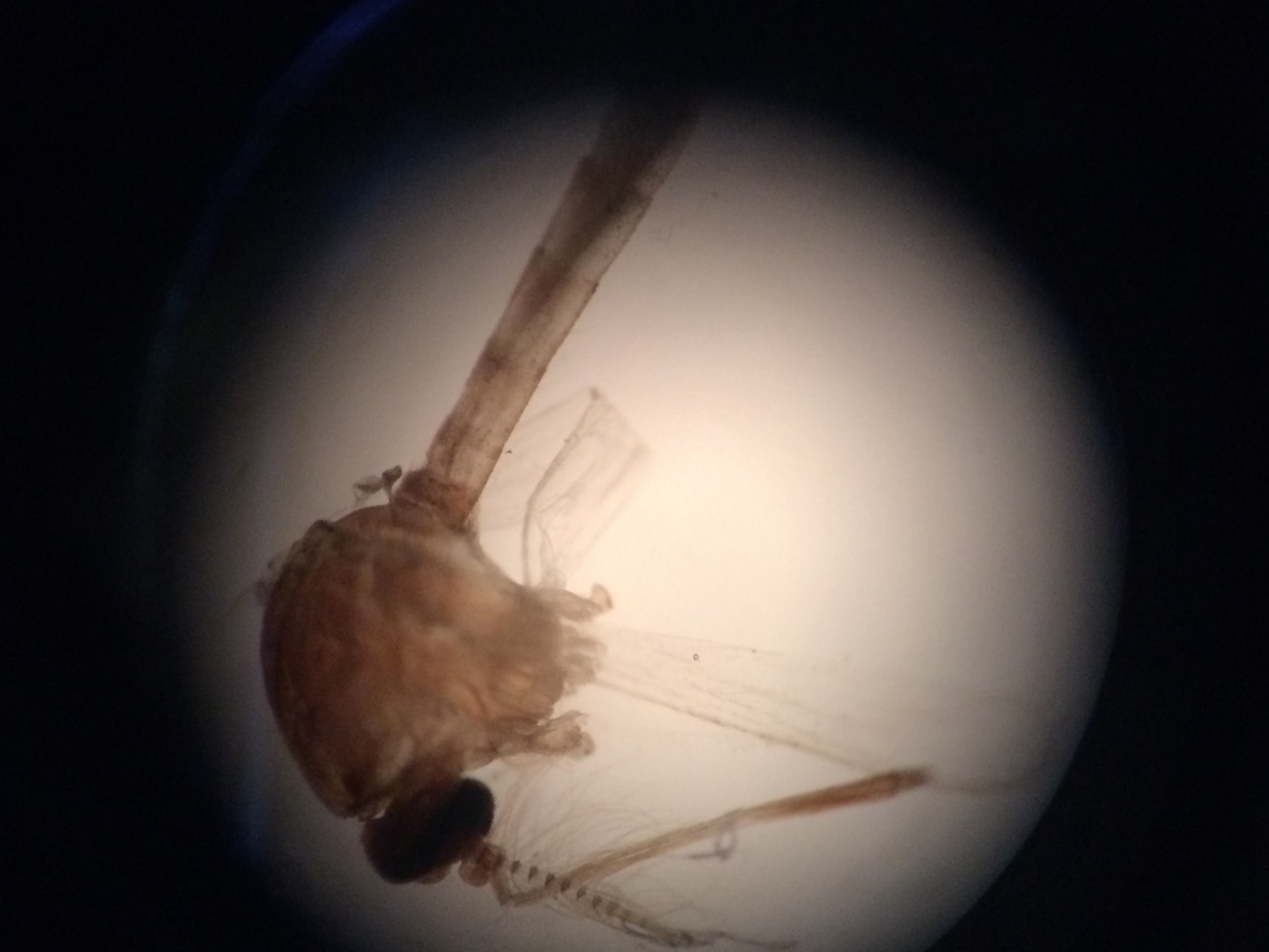


Fig. S3. *Culex theileri* whole body specimen.

Most features for *Cx. quinquefasciatus* Say, 1823 were identified as described by Jupp (1996). The specimen had distinct complete basal bands on the thorax with pale brown scutal scales. The ventral arm of phallosome extended laterally to the dorsal arm that is pointed at the apex (Jupp, 1996). The femora and tibiae did not have continuous white lines and had an entirely dark to dark brown proboscis. The wings had a short pale stripe of inner margin of costa near base with poorly ornate scutum and absent prelar scales (Jupp, 1996).


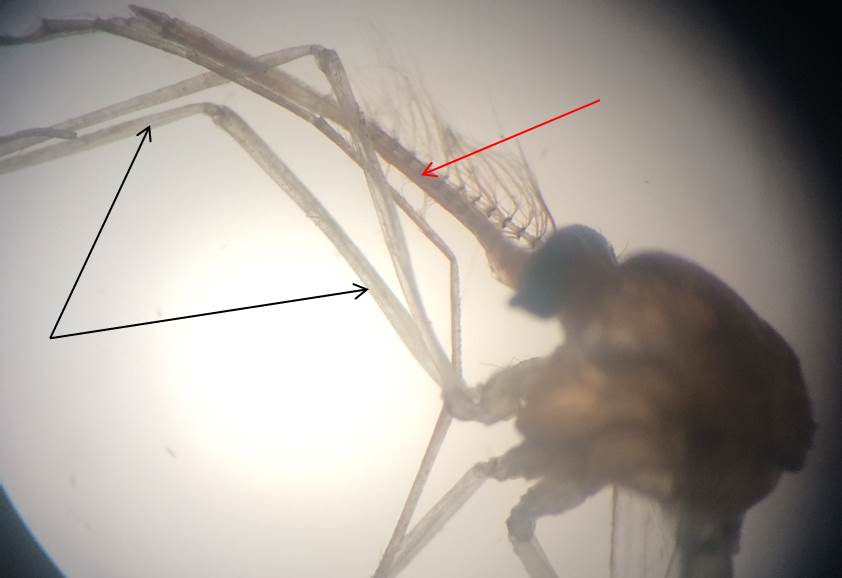


Fig. S4. Photo of the *Culex* *quinquefasciatus.* Red arrow highlighting the dark brown proboscis and black arrows showing femora and tibiae without continuous white lines.


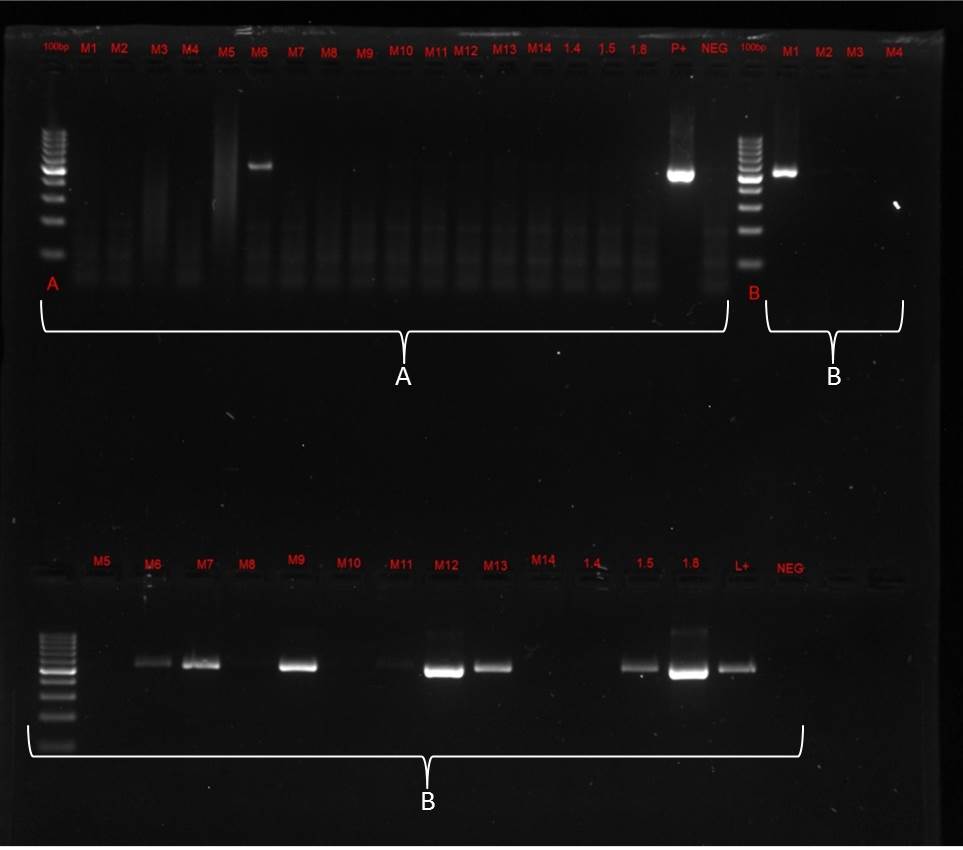


**Fig. S5. Agarose gel electrophoresis of amplified DNA from haemosporidian parasites using nested PCR amplification.** A) Samples M1- 1.8 (top), the amplification for *Plasmodium* sp. and B) Samples M1 – 1.8 (bottom) the amplification for *Leucocytozoon* sp. P+ = *Plasmodium* sp. control, L+ = *Leucozytozoon* sp. control, Neg = water control. A 100 bp O’Gene ladder was used to determine fragment size.


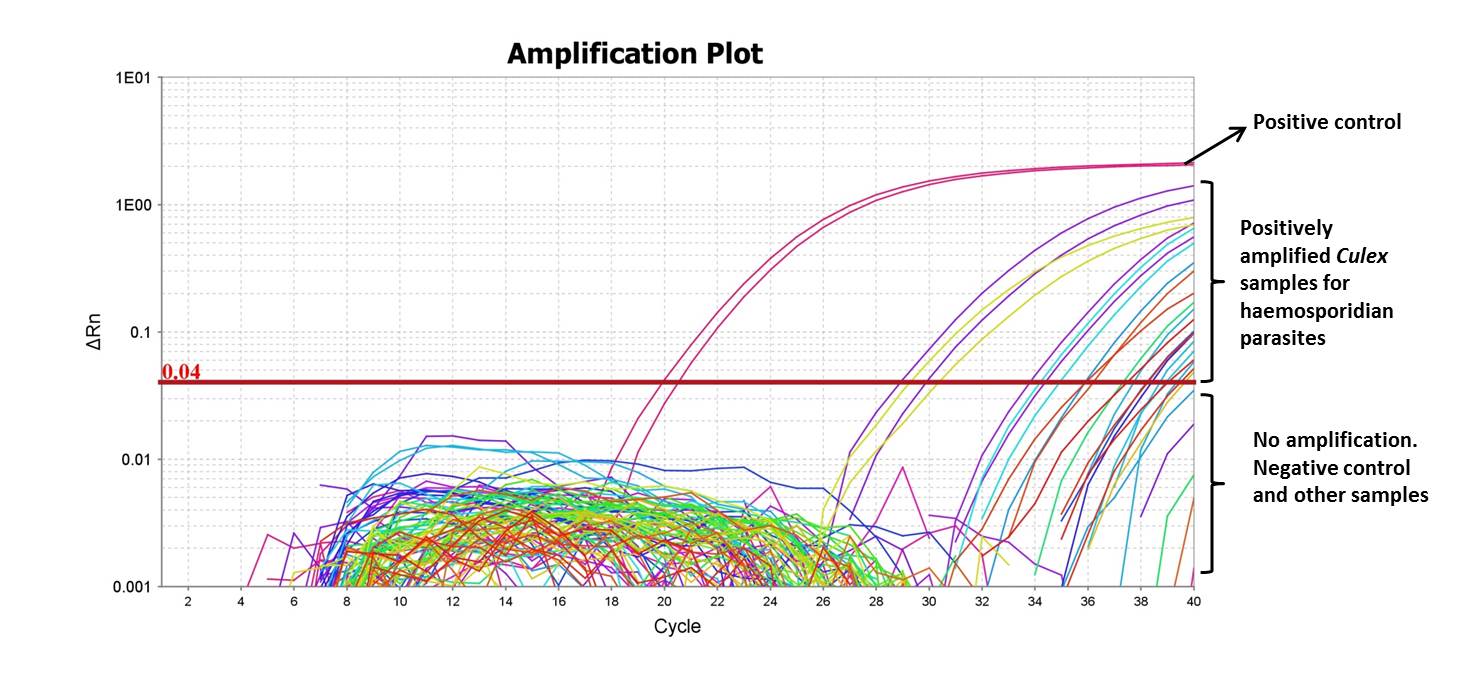


**Fig. S6. qPCR image of haemosporidian parasites showing the amplification peaks of positive mosquito samples**. gBlock of *Plasmodium* sp. was used as the positive control. Positive samples showed amplification above the threshold (0.04) and a quantitative cycle (<40).
